# Eye Tracking for Rehabilitation and Training in Paediatric Neurodevelopmental Disorders: A Systematic Review

**DOI:** 10.3390/brainsci16030337

**Published:** 2026-03-21

**Authors:** Guido Catalano, Sara Abbondio, Roberta Nicotra, Valentina Berselli, Marta Guarischi, Valentina Vezzali, Sabrina Signorini

**Affiliations:** 1 Department of Brain and Behavioral Sciences, University of Pavia, Via Agostino Bassi 21, 27100 Pavia, Italy; guido.catalano01@universitadipavia.it (G.C.); valentina.berselli01@universitadipavia.it (V.B.); 2 Developmental Neuro-Ophthalmology Unit, IRCCS Mondino Foundation, Via Mondino 2, 27100 Pavia, Italy; sara.abbondio@mondino.it (S.A.); valentina.vezzali@mondino.it (V.V.); sabrina.signorini@mondino.it (S.S.); 3 Unit for Visually Impaired People, Istituto Italiano di Tecnologia, Via Enrico Melen 82, 16100 Genova, Italy; marta.guarischi@iit.it

**Keywords:** eye-tracker, telerehabilitation, neurodevelopmental disorder, visual impairment

## Abstract

**Highlights:**

**What are the main findings?**

- Gaze-contingent eye-tracking interventions leverage the coupling between oculomotor control and fronto-striatal executive networks, producing improvements in attention, inhibitory control, social orienting, and visual processing in paediatric neurodevelopmental disorders.
- Eye-tracking systems modulate core neurocognitive functions (e.g., attentional control, joint attention, visuomotor integration) that cut across diagnostic categories.

**What are the implications of the main findings?**

- Targeting oculomotor–executive integration during developmental windows of heightened neural plasticity may enhance rehabilitation efficacy and early intervention strategies.
- Methodologically robust studies with standardised protocols and longitudinal designs are essential to clarify neural mechanisms and long-term transfer effects.

**Abstract:**

**Background:** Eye-tracking (ET) devices are gaining attention in technology-based paediatric rehabilitation through their intrinsic ability to assess patients’ engagement and visual attention within motivating, technology-based environments. We conducted a systematic review of available evidence from 2004 to 2025 on the implementation of ET in rehabilitative trainings targeting paediatric populations with neurological and neurodevelopmental disorders. This paper aims to outline the rehabilitative outcomes pursued in the clinical populations considered. **Methods:** This systematic review was conducted according to the Preferred Reporting Items for Systematic Reviews and Meta-Analyses (PRISMA) guidelines. Three electronic databases (PubMed, Web of Science, and Scopus) were consulted to summarise the state of the art of the last 20 years. Selected articles were categorised according to the type of treated disorder and the rehabilitated function. **Results:** ET devices have been increasingly integrated into paediatric rehabilitation with promising results across multiple neurodevelopmental conditions (e.g., ASD, ADHD, cerebral palsy). These systems have proven effective not only in training gaze control, but also in enhancing executive functions, social cognition, communication, and participation. Furthermore, they promote personalised and data-driven solutions and support high levels of engagement, feasibility, and user satisfaction. **Conclusions:** ET represents a promising frontier for paediatric rehabilitation, addressing various neurodevelopmental disorders. The gaze-contingent protocols employed have demonstrated potential effects in promoting adaptive behaviour across multiple developmental areas. Further research is warranted to provide shared guidance and to strengthen practice recommendations.

## 1. Introduction

Oculomotor control in infancy is foundational for visual development, as it enables infants to explore their environment, track moving objects, and interact with others. Through these processes, infants acquire visual, perceptual, and sensorimotor experiences that support the development of relational, cognitive, and motor milestones [1,2,3]. As a consequence, the presence of atypical oculomotor patterns represents a transnosographic condition in several developmental disorders, often associated with the atypical affective and behavioural patterns specific to various neurodevelopmental disorders [4]. Thus, children with autism spectrum disorder (ASD) commonly present oculomotor dysfunctions, including saccade dysmetria, difficulty in inhibiting saccades, and impaired tracking of moving targets [5]. Similarly, children with attention-deficit/hyperactivity disorder (ADHD) often exhibit abnormal saccadic eye movements, such as increased latency in anti-saccades and decreased accuracy in pro-saccades [6,7]. These oculomotor deficits are thought to contribute to the difficulties these patients experience in facial emotion recognition, social–affective tasks, and inhibitory control, as well as attention regulation [8]. Children with severe physical conditions often show impairments in motor, communicative, and relational skills, leading to a profound and multilevel disruption of their overall functioning with significant consequences in daily life participation [9]. In such cases, both in degenerative conditions (e.g., Rett syndrome [10]) and in non-degenerative developmental disabilities (e.g., cerebral palsy, CP), eye movements could represent a rehabilitative target and/or a relatively spared function. Among these conditions, CP represent a group of permanent neurological disorders affecting movement, balance, and posture, caused by damage or abnormal development in the brain, arising from non-progressive disturbances that occurred during a foetus or infant’s brain development [11]. Based on the predominant motor dysfunction, CP can be classified into spastic, dyskinetic, ataxic, and mixed forms. In spastic CP, ocular motor function is known to vary according to clinical severity and subtype, with most severe impairments typically observed in cases of quadriplegia [12]. Conversely, in children with dyskinetic CP, voluntary eye movements are often relatively preserved [13]. This residual eye-gaze function paves the way for its use in communication, education, leisure activities, and social interactions, offering new ways for these children to engage with their environment and the social context [14]. In early acquired/congenital visual impairment, atypical oculomotor patterns represent a common consequence of the visual deficit, often resulting in the presence of difficulties in fixation, smooth pursuit and saccades or abnormal ocular movements such as nystagmus or roving movements [15,16].

Given the pivotal role of oculomotor function in children’s development, the enhancement of this ability may constitute a valid goal for rehabilitation across various neurodevelopmental disorders, targeting their specific challenges and needs.

In recent years, the integration of new technologies in paediatric rehabilitation has expanded significantly. Digital and interactive tools, such as serious games or interactive training platforms, can support the development of highly engaging environments that promote active and focused patient participation [17,18,19]. In this context, the integration of eye-tracking systems represents a valuable tool for objectively assessing patients’ visual attention and engagement during technology-based rehabilitation tasks. Therefore, eye tracking has shown promising results for both communicative tools and the administration of neuropsychological assessments in patients with compromised verbal and/or motor skills. Eye-tracking technology provides objective and quantifiable physiological data on eye movement patterns, offering insights into mental processes and behaviour. These measurements are captured using eye trackers, digital devices that precisely record eye position and gaze direction over time, allowing researchers to analyse how individuals visually explore their environment. Eye-tracking data typically include different types of eye movements, such as fixations (periods in which the gaze remains relatively stable and visual information is processed) and saccades (rapid eye movements that shift gaze between points of interest). In some experimental paradigms, gaze-contingent techniques are used, in which the visual stimulus dynamically changes depending on the participant’s gaze position. Together, these measures allow researchers to investigate visual attention and cognitive processes and have shown increasing relevance in clinical neuroscience, particularly for understanding how individuals perceive and interact with their environment [20].

However, a systematic integration of eye tracking with traditional rehabilitation methods to improve oculomotor strategies in paediatric neurodevelopmental disorders remains underexplored. Hence, despite the growing interest in ET applications, there is limited understanding of gaze-contingent effectiveness for the above-mentioned paediatric populations, particularly regarding their feasibility and efficacy in improving oculomotor competences.

This review aims to focus on the use of ET devices as tools to support rehabilitative interventions in heterogeneous paediatric clinical populations. By summarising the available evidence, the review seeks to provide an up-to-date and critical appraisal of the advantages and limitations of this approach, highlighting its potential in enhancing rehabilitation outcomes and identifying gaps in the existing body of research.

## 2. Materials and Methods

### 2.1. Search Strategy

A systematic search was performed across three electronic databases: PubMed, Web of Science, and Scopus. The search included studies published in the last 20 years (from January 2004 to August 2025) in English or Italian. The following Boolean string was used: (“training” OR “oculomotor training” OR “oculomotor” OR “rehabilitation” OR “game” OR “gaming”) AND (“eye tracking” OR “eye tracker” OR “eye pointer” OR “eye gaze”) AND (“children” OR “infant*” OR “adolescent*” OR “pupil*”).

Additional references were identified through manual search of bibliographies of included articles and previous reviews. Details of the search strategy and terms used for each database are reported in Supplementary Table S1.

### 2.2. Eligibility Criteria

Randomised controlled trials, clinical trials, protocols for feasibility studies, and single-case studies investigating the use of ET systems for training or rehabilitation in paediatric developmental disorders were included. The populations of interest were children and adolescents (0–18 years old) affected by neurological, neurodevelopmental and visual disorders (e.g., ASD, ADHD, learning disorders, cerebral palsy, amblyopia, genetic syndromes). Only studies adopting gaze-contingent ET protocols for training where eye movements were used to trigger events on the screen in response to the participant’s gaze were included, such as gaze-controlled serious games, gaze-driven attention trainings, and dichoptic games. Conversely, studies were excluded if ET techniques had been addressed only as a tool to either measure treatment outcomes or provide feedback signals in a closed-loop system (e.g., in vestibular treatment). Moreover, ET-based protocols that were developed in non-clinical contexts, such as sport training, workplace evaluation, or non-rehabilitative medical fields (e.g., surgery trainings or procedures), were excluded.

### 2.3. Selection Process

All records were independently screened by two reviewers (VB, SA) using the Rayyan QCRI platform [11]. First, titles and abstracts were reviewed; then, full texts were assessed for eligibility. Disagreements were resolved by a third reviewer (RN). Reference management was performed using Mendeley Desktop (the Mendeley Cite v1.69.3).

### 2.4. Data Extraction and Synthesis

For each included study, data were extracted on the following: author(s), year, study design, sample size and characteristics, clinical condition, intervention type and duration, outcome measures, and main results. Data were organised in a summary table (see Appendix A). Narrative synthesis was performed due to high heterogeneity in populations, interventions, and outcomes. Due to variability in study designs and reporting standards, a formal meta-analysis was not feasible.

## 3. Results

### 3.1. Study Selection

**Figure 1:**
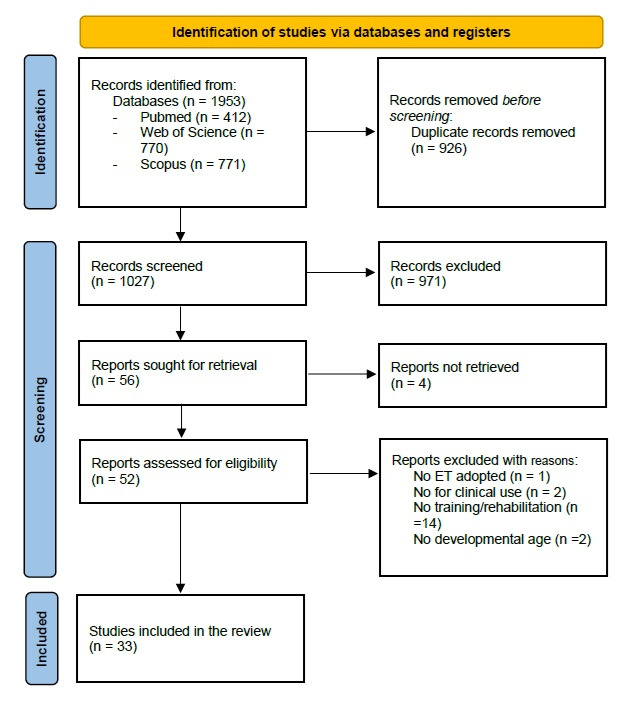
PRISMA flowchart.

After data extraction, 33 studies were selected for this review, all involving paediatric participants (0–18 years) with neurodevelopmental or neurological conditions. The PRISMA flowchart detailing the identification and selection process is presented in Figure 1. The PRISMA checklist is available as Supplementary Material.

### 3.2. Clinical Populations and Study Designs

**Figure 2:**
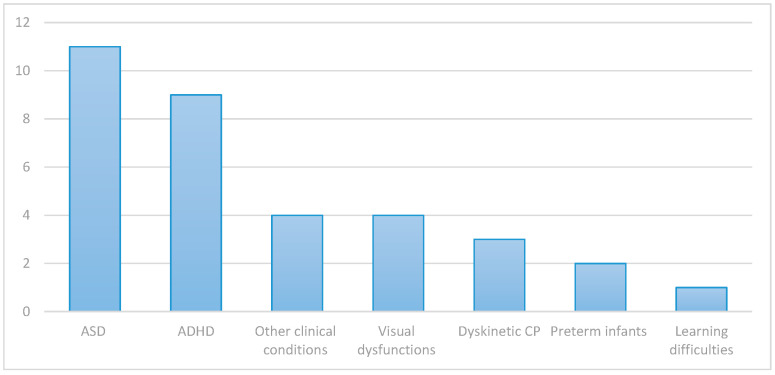
Number of papers included per treated disorder. This column chart outlines the number of papers included for each disorder. ASD (*n* = 11), ADHD (*n* = 9), dyskinetic cerebral palsy (*n* = 3 papers), learning difficulties (*n* = 1), visual dysfunctions (*n* = 4; includes “amblyopia” and “low vision” categories), preterm infants (*n* = 2), and other clinical conditions (*n* = 4; includes “Rett syndrome”, “Fragile X Syndrome”, “special educational needs”, and “physical impairments” categories). The total number of papers in the figure is 34, as one addresses two populations (ASD and ADHD patients).

The selected studies encompassed a heterogeneous range of clinical conditions (see Figure 2), including attention-deficit/hyperactivity disorder (ADHD, *n* = 9), autism spectrum disorder (ASD, *n* = 11), dyskinetic cerebral palsy (CP, *n* = 3), Rett syndrome (*n* = 1), amblyopia (*n* = 2), and low vision (*n* = 2). Additionally, a few studies focused on very preterm infants (*n* = 2), learning difficulties (*n* = 1), children with Fragile X Syndrome (*n* = 1), or other neurodevelopmental profiles (e.g., special educational needs and physical impairments, *n* = 2). The resulting sum is 34, as one paper [21] addressed two populations (ASD and ADHD patients).

Methodologically, most of the studies employed a variety of designs, including randomised controlled trials, pilot or feasibility studies, or clinical trials. A smaller subset employed single- or multiple-case designs. In addition to the previously mentioned studies, four study protocols [22,23,24,25] were also included, in order to widen the description of possible applications of ET for clinical purposes in developmental disorders. Most studies were conducted in clinical or research settings, though a significant portion adopted home-based interventions [22,23,26,27,28].

### 3.3. Intervention Characteristics

Across the reviewed studies, eye-tracking (ET) devices were employed in various rehabilitative interventions. The common element was the use of gaze-contingent systems, whereby the child’s eye movements directly triggered or modulated events within interactive environments. The majority of studies reported qualitative indicators of feasibility, including high levels of engagement, adherence, and positive feedback from parents or caregivers.

ET-based interventions were highly heterogeneous, including gaze-contingent serious games targeting inhibitory control (e.g., go/no-go paradigms), interactive environments such as virtual hide-and-seek tasks, and structured training modules in which children were required to maintain fixation on targets while ignoring distractors, with progressively increasing difficulty levels [26,29,30].

Most interventions [24,26,31,32,33] aimed to improve executive functioning (e.g., attention, inhibitory control, impulse control), cognitive abilities and learning abilities such as memory, and cognitive flexibility [21,27,30,34,35,36]. Others aimed to enhance visual outcomes (e.g., visual acuity, stereoacuity) [37,38,39,40], social cognition and gaze behaviour (e.g., eye contact, gaze direction, joint attention) [25,41,42,43,44,45,46]. Studies addressing more severe conditions had a specific focus on supporting and/or facilitating patients’ communication and participation [14,39,47,48]. Finally, only one study focused on promoting fine visuomotor coordination competences [33]. The duration of interventions ranged from brief, intensive two-week protocols to more extended programmes lasting up to eight months. Delivery formats included home-based gaming, school-based cognitive training, and structured telerehabilitation protocols, demonstrating the flexibility and adaptability of ET devices across settings.

### 3.4. Functional Domains Targeted by Interventions

The following section describes the most targeted domains in the included interventions. Table 1 reports which domain has been targeted for each condition. Functional domains were defined pragmatically based on the primary targets of the interventions reported in the included studies. Because many eye-tracking-based training paradigms simultaneously target attentional regulation and executive control processes (e.g., inhibitory control or goal-directed gaze behaviour), attention and executive functions were grouped within a single domain for descriptive purposes.

#### 3.4.1. Attention and Executive Functions

The most robust evidence emerged in the domain of attentional control and executive functioning, particularly among children with ADHD. Several studies reported improvements from the use of ET-based interventions in sustained attention, reaction times, and inhibitory control. For instance, García-Baos et al. [28] observed significant gains in fixation, reaction time and impulsivity after a three-week home-based game, while Lee et al. [31] and Rudolf et al. [33] respectively documented improved inhibitory control and focused attention. Moreover, home-based training with ET has been demonstrated to be useful in promoting visuospatial attention in school-aged children with ASD [49]. The review also included two study protocols: one targeting children at familial risk of ADHD [23] and the other one targeting very preterm (VP) infants [24]. Moreover, one RCT [32] investigated the feasibility of Attention Control Training in very preterm infants, showing promising results in domains related to attentional regulation.

#### 3.4.2. Cognitive and Learning Enhancement

Several interventions were designed to enhance broader cognitive and learning-related abilities such as memory processes and learning speed in children with neurodevelopmental disorders.

Chan and colleagues [21,34] showed substantial improvements in learning speed and visuospatial working memory in children with ADHD, ASD, and learning difficulties. Janmohammadi et al. [30] and Garcia-Zapirain et al. [35] also reported that training in gaze control and visual pursuit led to behavioural regulation and faster learning in children with attention problems. Eye-gaze training has also been used to test reading ability improvements in nonverbal children with special educational needs by Arnold and colleagues [36]. However, the results did not provide significant evidence for treatment efficacy.

#### 3.4.3. Social Cognition and Gaze Behaviour

In ASD populations, many studies focused on improving sensitivity to social cues, eye contact, and emotional processing through ET-based interventions. Sosnowski et al. [50] developed a gaze-contingent video game combining applied behaviour analysis with ET, reporting improved emotion recognition post-intervention. Other approaches implemented self-monitoring and reinforcement strategies to enhance spontaneous eye contact in children with ASD, obtaining significant evidence of training feasibility and efficacy [41,45]. A randomised controlled trial was conducted by Tang and colleagues [46] to investigate the effects of storytelling with social contextual information in improving visual attention and gaze direction in children with ASD. The results show that storytelling with social contextual information improved gaze behaviour toward faces or eyes in ASD and TD when both were assessed with photos displayed on a screen. Wang et al. [51] provided evidence that even toddlers at risk for ASD can benefit from gaze-based social attention training, particularly in directing gaze toward faces.

Additionally, ET training has been implemented with Virtual Reality (VR) systems to enhance gaze fixation in ASD children [29,42], respectively showing potential efficacy of the approach and significant positive results in subgroup comparison. Social cognition and gaze behaviour have also been investigated in other conditions, such as Fragile X Syndrome [44] and dyskinetic cerebral palsy [43], respectively showing improvements in social gaze and oculomotor performances after the training.

#### 3.4.4. Communication and Participation

Concerning patients with severe physical impairments, especially children with dyskinetic CP and Rett syndrome, ET devices were employed as assistive technologies to facilitate interaction, autonomy, and participation. Several studies [14,47,48] demonstrated increased goal achievement and sustained use of gaze-based systems for communication in daily life. Notably, Puttemans et al. reported positive outcomes across multiple metrics including psychosocial impact, autonomy, and heart rate variability. Another study [27] showed promising preliminary results after remote eye-tracking training in facilitating access to telerehabilitation and remote school tasks for girls with Rett syndrome.

#### 3.4.5. Vision Rehabilitation

In children with amblyopia or low vision, ET training has been proposed as an alternative or supplement to conventional therapies such as patching. Two studies [37,38] found that gaze-based dichoptic interventions were comparable in efficacy to traditional occlusion therapy, with better adherence and acceptability. Additionally, Donmez & Cagiltay [28,39] developed ET-supported visual training games that were well-received by children with visual impairments, supporting their potential in remote or school-based rehabilitation. Lee and colleagues [40] investigated the effectiveness of computerised eye-tracking training in improving saccadic eye movements in children with ADHD, obtaining significant results in both saccade latency decrease and saccade accuracy after ET training.

## 4. Discussion

### 4.1. Principal Findings

This review specifically addressed the use of eye-tracking devices as rehabilitative tools in paediatric populations, positioning its scope in the broader field of gamified and technology-enhanced neurorehabilitation in childhood [52]. It demonstrates that ET devices have been increasingly integrated into paediatric rehabilitation, with promising results across multiple neurodevelopmental conditions. In some studies, oculomotor functions (e.g., fixation stability or saccadic control) represented the direct target of the training, whereas in others, eye movements were primarily used as a tool to support the rehabilitation of higher-level cognitive or social functions. Thus, although heterogeneous in design and target populations, the included studies demonstrate that gaze-contingent systems can enhance multiple functional domains: attention and executive functions, social cognition, communication, and participation. The strongest concentration of evidence emerged for autism spectrum disorder (ASD) and attention-deficit/hyperactivity disorder (ADHD), likely reflecting both the prevalence and the concurrent large body of literature regarding such conditions and the suitability of ET-based paradigms for supporting their core deficits.

Most studies reported high levels of engagement, feasibility, and user satisfaction, especially in home-based or gamified settings, even in infants and children with severe disabilities, with parents reporting positive impacts.

To provide a coherent narrative, the discussion is organised with focus on the functional domains targeted by rehabilitative interventions. This structure reflects the fact that ET-based interventions operate on core mechanisms (e.g., attentional control, social orienting, visuomotor behaviour, and communication), which cut across multiple neurodevelopmental conditions and align more closely with rehabilitation goals than categorical diagnoses.

### 4.2. Eye Tracking as a Tool for Neuropsychological Rehabilitation and Learning Enhancement

In children with neurodevelopmental conditions (e.g., ADHD, ASD, learning difficulties) or mixed developmental profiles, ET-based training has been associated with measurable improvements in focused attention and inhibitory control as well as in learning speed, visuospatial working memory and flexible thinking. For example, some gaze-contingent paradigms require the child to suppress reflexive saccades toward salient stimuli and instead direct gaze toward task-relevant targets, thereby training inhibitory control and oculomotor function. These findings align with evidence that top-down control of gaze is tightly coupled with the maturation of fronto-striatal circuits in the regulation of visual attention and executive function. In fact, cortico-striatal projections—which have their nuclei in the frontal lobe—are the neurophysiological substrates of goal-directed and habitual behaviour as well as executive functioning [53]. From a neurophysiological perspective, voluntary control of eye movements relies on prefrontal and striatal systems [7,54]. These systems govern the ability to inhibit automatic responses and orient attention in accordance with task demands. Research about oculomotor function in ADHD confirms deficits in response preparation and inhibitory control, independent of symptom subtype [55]. The developmental perspective supports this view: voluntary oculomotor control matures slowly through adolescence in parallel with synaptic pruning and myelination in fronto-parietal networks [1]. This protracted plasticity window provides an opportunity for interventions targeting oculomotor–executive integration as it could happen when introducing ET-contingent trainings, even in short-term settings [26].

Some ET interventions were also implemented in home-based settings [22,23,26,27,28], suggesting the potential feasibility of delivering gaze-contingent training outside traditional clinical environments. Bringing such an approach directly to families of children with complex disabilities or neurodevelopmental disorders holds promise in supporting the continuity of care and improving access to rehabilitation services. In line with this perspective, previous research has suggested that telerehabilitation can represent a promising therapeutic approach, particularly when in-person rehabilitation is difficult to deliver, with outcomes that may be comparable to or even better than conventional conditions [52].

The current literature about the use of ET as a tool for neuropsychological rehabilitation also focuses on prevention, providing study protocols addressed to “at-risk” populations regarding cognitive and executive functioning [23,32], suggesting that ET may one day serve as not only rehabilitation but also early cognitive/executive function scaffolding.

### 4.3. Eye Tracking as a Tool Aimed at Enhancing Social Cognition and Communication in Neurodevelopmental Disorders

A large portion of ET research has targeted autism spectrum disorder (ASD), focusing on emotion recognition, attention to faces, joint attention, and spontaneous eye contact, which represent most of the ASD core symptoms [56]. This is grounded in evidence that children with ASD show atypical visual exploration patterns characterised by: reduced prioritisation of faces and socially informative regions, decreased sensitivity to gaze cues, increased reliance on low-level visual saliency, and a stronger centre bias. These peculiarities are related to the disruption of joint attention and Theory of Mind [57]. Indeed, accurate gaze direction has been considered the milestone of the social cognition system, and joint attention can be considered a goal-directed behaviour whose primary aim is to share experience and wills with other people [58,59]. Therefore, children with ASD show—together with disruptions of social–emotional processing, social communication and Theory of Mind (ToM)—a significant early impairment of eye-gaze competencies for social purposes [59].

The analysed ET-based interventions attempt to “re-shape” these visual strategies by guiding the child toward socially meaningful stimuli, including faces, eyes, and emotional expression [46,50,51], even in combination with self-monitoring and reinforcement strategies, obtaining significant results [41,45].

Research also provides evidence for the enhancement of gaze fixation when combining ET training with Virtual Reality devices [29]. VR-ET approaches may offer rich and highly controlled environments in which clinicians can systematically manipulate the complexity, predictability, and saliency of social stimuli while capturing real-time gaze metrics, thus balancing experimental control with ecological validity [60]. Moreover, it has been suggested that stimulus characteristics, such as modality and familiarity, may influence gaze behaviour in children with ASD, potentially enhancing attention to socially relevant cues such as faces [61].

### 4.4. Eye Tracking as a New Avenue for Participation in Complex Motor Disorders

For children with severe motor and/or cognitive impairments—such as Rett syndrome and dyskinetic cerebral palsy—ET devices may represent a meaningful supplement in the enhancement of traditional tools to support communication and everyday functioning, offering a direct access pathway to digital interaction.

In common clinical practice, aided augmentative and alternative communication (AAC) is an effective means to supplement and enhance the functional communication skills of individuals with communication and language impairments [62,63]. AAC is appropriate whenever a person’s speech cannot meet their daily communication needs. However, in the considered populations, limited hand use or speech may limit the usability of such a tool. In the selected works for this review, ET-based assistive technology emerges as a potential enhancer of communication, choice-making, and exploration in ways that traditional interfaces, such as AAC, do not.

The studies included in this review consistently reported improvements in functional goal attainment, autonomy, and sustained usage over time and qualitatively reported increased motivation and enjoyment, and reduced frustration for both children and caregivers [14,27,47,48]. These findings position ET not only as a rehabilitative tool but also as a compensatory access modality that bridges motor, communicative, and cognitive barriers.

### 4.5. Eye Tracking for Visual Function Rehabilitation

Eye-tracking devices represent an emerging tool for the rehabilitation of visual functions, both in conditions primarily affecting the visual system and in neurodevelopmental disorders where oculomotor control is impaired. Across the studies included in this review, two main applications can be distinguished: (1) treatments targeting primary visual disorders, such as amblyopia or low vision; and (2) interventions aimed at improving oculomotor control in neurodevelopmental disorders such as ADHD, ASD, and cerebral palsy.

Among ET-based interventions targeting visual disorders, amblyopia represents the condition with more applications. Although occlusion therapy remains the gold-standard treatment for amblyopia, gaze-contingent approaches should be interpreted within the broader framework of the perceptual learning paradigm, which aims to enhance visual performance through repeated practice on contrast, spatial frequency, and binocular integration tasks [64].

With technological advancements, this paradigm has evolved toward interactive and game-based formats designed to stimulate the amblyopic eye while maintaining engagement. According to available studies, eye-tracking systems seem to be integrated into this model by enabling real-time monitoring of fixation stability, binocular alignment, and gaze-contingent stimulus presentation [37,38]. In contrast to passive occlusion, ET-driven tasks require continuous visual interaction, potentially reinforcing active perceptual processing and binocular cooperation. Moreover, gamification may enhance adherence, which is a well-known limitation of traditional patching therapy.

Importantly, visual functions are rarely isolated from cognitive, attentional, or motor systems. As discussed above, several neurodevelopmental disorders included in this review, ADHD, ASD, and dyskinetic CP, are known to present abnormalities in oculomotor control, such as impaired fixation stability, atypical saccadic patterns, or reduced ability to orient attention. ET-based interventions may therefore target visual skills that indirectly support cognitive or motor rehabilitation, as shown in several included studies [30,40,43,50].

### 4.6. Limitations of Current Research

Despite the encouraging results, this study highlighted some limitations in the current literature about ET-based rehabilitation protocols. First, many studies rely on small and heterogeneous samples, which limit the generalisation of findings. Moreover, the duration of the interventions is often short, lacking follow-up assessments to evaluate long-term effects. Another critical issue is the absence of standardised intervention protocols, which does not enable the reproducibility and comparability of results across studies. In addition, technical details are frequently underreported, particularly regarding calibration accuracy and overall data quality. Finally, outcome measures are often based on parent-report or proxy-rated scales, which may introduce subjectivity and bias when evaluating intervention effectiveness.

### 4.7. Future Directions and Clinical Implications

Future research should broaden the range of disorders targeted by ET rehabilitation. For example, Cerebral Visual Impairment offers several critical rehabilitative goals targeted by ET, such as oculomotor and cognitive/executive dysfunction [65,66]. Further research is warranted to consolidate their role in clinical practice and to strengthen practice recommendations. Specifically, future studies should focus on the design and implementation of multicentre randomised controlled trials (RCTs) with adequate statistical power to ensure reliability and generalisability of findings. Moreover, it will be important to investigate the longitudinal effects of the interventions and their applicability in real-world settings, providing evidence for their long-term efficacy and ecological implementation in rehabilitation. Efforts should also concern the development of standardised training paradigms, useful for cross-study comparisons to define best practices.

## 5. Conclusions

Eye-tracking devices represent a novel and multifaceted platform for paediatric rehabilitation. Gaze-contingent protocols showed significant potential effects in enhancing neuropsychological functions, learning, social and communication skills, oculomotor performance and autonomy in various disorders. Finally, the integration of wearable ET systems and mobile applications could represent another promising direction as it makes way for more ecological interventions.

## Figures and Tables

**Table 1:** Framework of the clinical domains investigated in each condition. ASD: autism spectrum disorder; ADHD: attention-deficit and hyperactivity disorder; CP: cerebral palsy; EFs: executive functions; v-m: visuomotor.

	Attention and EFs	Cognitive and Learning	Social Cognition	Participation and Communication	Visual Function	Fine v-m Coordination
ASD	X	X	X		X	
ADHD	X	X			X	X
Dyskinetic CP				X	X	
Learning difficulty	X	X				
Preterm infants	X	X	X			
Other complex disabilities		X		X	X	
Visual dysfunctions				X	X	

X indicates which domain has been targeted for each condition.

## Data Availability

No new data were created or analysed in this study.

## Supplementary Materials

The following supporting information can be downloaded at https://www.mdpi.com/article/10.3390/brainsci16030337/s1. Table S1: Search builders according database, limited for the last 20 years of publication. File S1: PRISMA 2020 checklist [67].

## Appendix A. Highlights of the Included Studies

**Reference/Study and Year**	**Study Design**	**Population**	**Primary Outcome**	**Outcome Measure**	**Intervention**	**Main Results**
[36] Arnold et al. (2025)	Clinical trial (experiment 2)	33 participants (mean age: 142 months) with special educational needs	To evaluate whether eye-gaze training prior to use in the digitised reading test would improve word recognition scores for nonverbal children	WISC-IV; modified word recognition test with eye-gaze facility (EG-MNSRT)	Two online eye-gaze games presented in 10 min slots	No significant differences between the reading performance of those who did and did not participate in eye-gaze training and no impact of diagnosis, verbal IQ, perceptual reasoning IQ, or receptive vocabulary ability
[43] Bekteshi et al. (2020)	Clinical trial	10 children (4.4–13.6 years) with dyskinetic cerebral palsy	To investigate the operational competences screen navigation and dwell function underlying eye-gaze performance and the relation of dystonia and choreoathetosis with eye-gaze performance in children with dyskinetic cerebral palsy	Task performance (sum of targets reached in 90 s); fixation count (total number of eye-gaze fixations in 90 s); eye movement accuracy	6 video games in a standardised order and a duration of 90 s for each game, chosen from the Sensory Eye Fx (Tobii Dynavox, Danderyd, Sweden)	Significant improvement in eye-gaze task performance and within-subject correlations between eye movement accuracy and task performance and between fixation count and eye movement accuracy
[14] Borgestig et al. (2017)	Non-experimental multiple-case study (before, after and follow-up design)	10 children (1–15 years) with severe physical impairments without speaking ability	To establish the impact of a gaze-based assistive technology (AT) intervention on activity repertoire, autonomous usage, and goal attainment in children with severe physical impairments	Computer usage diaries; Goal Attainment Scaling (GAS)	9–10-month intervention with eye-gaze ET and assistive technology software with different tasks and activities	All sustained usage in daily activities and all attained goals. Proved efficacy in guiding parents and teachers to support children performing activities with the AT
[42] Mei et al. (2018)	Conference paper	10 children with ASD	To investigate the effectiveness of an approach using a Customisable Virtual Human (CVH) and Virtual Reality (VR) compared to NCVH conditions in training joint attention in children with ASD	Reaction time of joint attention and time gazing at regions of interest (ROIs)	To play the with Customisable Virtual Human (CVH) device with a VR game interface combined with ET	CVH may be more effective in preventing atypical attention patterns that are common in ASD
[29] Yu et al. (2024)	Randomised controlled trial	24 children with ASD. Mean ages: control group (CG)—3.71 (0.75); avatar-uncustomised group (AUG)—6.17 (1.26); avatar-customised group (ACG)—4.24 (1.80)	To evaluate the effectiveness of the Hide-and-Seek Virtual Reality System (HSVRS) in improving gaze fixation among children with ASD	Subjective questionnaires completed by the participants’ parents and objective eye-gaze data	5 repeated trials over a period of 7 days. Participants were asked to play a game of hide and seek using our HSVRS	No significant differences in the questionnaire scores between the 3 groups. Significant improvements in two-group comparison (AUG-CG, ACT-CG). Significant difference in face fixation proportion and background fixation proportions between the AUG and the ACG
[34] Chan et al. (2022)	Randomised controlled trial	53 primary school students with learning difficulty/neurodevelopmental disorders (experimental group, *n* = 27; control group, *n* = 26)	To examine the effects of eye-tracking training as an after-school programme to enhance learning and memory	Matrix Reasoning and Similarities subtests of the Wechsler Intelligence Scale for Children-Fourth Edition; Hong Kong List Learning Test (HKLLT); Chinese Word Reading Test; Chinese Passage Read	Six modules of ET training, each trial lasting 5 or 10 min. 20 weekly sessions of after-school training, 50 min per session, for 8 months	Improved memory and faster learning in the experimental group. Students with poorer learning scores at baseline demonstrated a greater degree of improvement in learning
[21] Chan et al. (2024)	Clinical trial	40 students (6–11 years) with neurodevelopmental disorders (experimental group, *n* = 20; control group, *n* = 20)	To explore whether after-school eye-tracking training can improve visuospatial working memory (VSWM) and cognitive flexibility performance	Digital visual memory span test (D-VMST) to assess VSWM and Five-Point Test to assess cognitive flexibility	Six modules of ET training aimed at improving attention and inhibitory control. Each trial of training lasts 5 or 10 min	Significant improvement in VSWM and flexible thinking in the experimental group
[49] Chukoskie et al. (2018)	Clinical trial	8 children (mean age: 13.9) with ASD	To demonstrate the feasibility of home-based training with an ET	Spatial Attention Task (E-Task), Gap–Overlap Saccade Task	8-week training (30 min per day, 5 times per week). Games on a laptop with ET designed with principles to train fixation, speed and accuracy of eye movements and control of visuospatial attention	Feasibility of training is demonstrated
[45] Demirtas et al. (2025)	Multiple-probe study	3 children (12–13 years) with ASD	To evaluate the effectiveness of a self-monitoring strategy intervention package on eye contact performance of children with autism spectrum disorder (ASD) through self-monitoring, goal setting, and reinforcement	Visual analysis and Tau-U	Participants wore eye-tracking devices and used self-monitoring to observe their eye movements during a 5 min face-to-face conversation, 5 days a week during school hours. Four 5 min sessions a day	The intervention package significantly improved participants’ eye contact performances
[39] Donmez & Cagiltay (2019)	Single-subject case study	One subject with low vision (age not specified)	To present the design and development procedure of three prototype eye movement games and a final product enhanced with the experience from the prototype ones for students with low vision	Qualitative interview	Three prototype eye movement games and a final product game supported by ET	The subject enjoyed the study and the serious games
[28] García-Baos et al. (2019)	Randomised controlled trial	28 children with ADHD (8–15 years)	To assess whether the eye-tracking approach of the RECOGNeyes game has potential therapeutic benefits for children with ADHD	Frog Task Word recognition task	RECOGNeyes with eye tracker for 3 weeks (3 times a week) at home	Participants from the ET group showed significant improvement in impulsivity, reaction time, and fixation gaze control
[35] Garcia-Zapirain et al. (2017)	Clinical trial	19 children (mean age: 10.88 years) with ADHD	To develop and test a dual system for the rehabilitation of cognitive functions in children with ADHD, an arithmetical game-based application that makes use of an eye tracker and a hand tracker for interaction with it	SUS questionnaire, QUIS questionnaire	The system is made up of the ET, the gesture recognition device on one side and then the client side. On the client side, the user interacts with a Natural User Interface by using sensors	Globally quite positive user evaluation of the proposed multimodal rehabilitation system
[23] Goodwin et al. (2016)	Study protocol	50 infants (10–14 months) at familial risk of ADHD	To test the potential efficacy of the INTERSTAARS cognitive trial	Eye-tracking, observational, parent-report and neurophysiological measures	9 weekly home-based attention training sessions targeting attention control with gaze-contingent animations via ET technology	Study protocol
[44] Hall & Britton (2024)	Clinical trial	65 patients with Fragile X Syndrome, FXS (*n* = 33), or non-syndromic autism (*n* = 32)	To examine potential differences in social learning between individuals with FXS and individuals with non-syndromic ASD by administering the behavioural treatment probe devised by Gannon et al.	Heart rate; social gaze	Blocks of looking while listening trials alternated with blocks of looking while speaking trials with reinforcing method (token)	Improvements in social gaze following the treatment probe for males with FXS; improvements in social gaze more modest for males with non-syndromic ASD; no effect of the treatment probe on heart rates in either group
[26] Iannizzotto et al. (2020)	Clinical trial	12 girls at late primary and secondary school ages with Rett syndrome	To investigate new ways to facilitate access to eye-gaze-based interaction for the specific case of simplified communication and to investigate the efficacy of SWYG software for telerehabilitation and remote school tasks	To answer 8 questions by means of the eye gaze	Remote eye-tracking architecture composed of SWYG (Speak With Your Gaze) and Video Conferencing System (VCS)	Promising preliminary results
[30] Janmohammadi et al. (2020)	Randomised controlled trial	39 boys (6–10 years) with ADHD (experimental group, *n* = 20; control group, *n* = 19)	To determine the efficacy of a visual tracking/pursuit paradigm based on visual fixation (gaze) and head control, and control of eye movement speed, in addition to conventional therapy for ADHD	Conner’s Parent Rating Scale; Continuous Performance Task-2; Test of Visual-Motor Skills-Revised	A 5-week (2 sessions per week, 30 min per session) ET intervention based on the isolation of neck and eye movement	The training led to decrease in and inhibition of unwanted saccadic eye movements. Visual tracking interventions could modify behaviour (Conner’s Parent Rating Scale results)
[47] Karlsson et al. (2019)	Clinical trial	5 subjects (3–5 years) with dyskinetic cerebral palsy	To trial two eye-gaze control devices in goal achievement, communication, and participation outcomes	Preschool Language Scale-4 (PLS-4); Focus on the Outcomes of Communication Under Six (FOCUS©); Young Children’s Participation and Environment Measure (YC-PEM); Cerebral Palsy Quality of Life Questionnaire—Child version; Dimensions of Mastery Questionnaire (DMQ); Canadian Occupational Performance Measure (COPM); Goal Attainment Scale (GAS); Responsive Augmentative and Alternative Communication Style Scale Version 3 (RAACS)	Two eye-gaze control technology systems, each for 6 weeks, with an intervening 6-week washout period and an assessment at the end of each period	Improvements in goal achievement (GAS, COPM) and performance
[31] Lee et al. (2021)	Clinical trial	32 children (6–12 years) with ADHD (experimental group, *n* = 16; control group, *n* = 16)	To examine the effect of an eye-tracking training programme on inhibitory control in children with ADHD	Conners’ Parent Rating Scale-Revised: Short Form; Eriksen Flanker Test; Cantonese version of Category Fluency Test; Five-Point Test; Children’s Color Trails Test	Six modules (10 min each) of ET training aimed at improving attention and impulse control	Significant improvement in inhibitory control in the experimental group after training
[40] Lee et al. (2020)	Pilot study	18 participants (6–12 years) with ADHD (experimental group, *n* = 9; control group, *n* = 9)	To investigate the effectiveness of computerised eye-tracking training on improving saccadic eye movements in children with ADHD	Saccade latency and accuracy in an anti-saccade task and in a pro-saccade task before and after training	ET training aimed at improving inhibitory control, mental flexibility, and attention. 2 weeks of 8 training sessions, 10 min each	Significant decrease in saccade latency (anti-saccade task) and significant increase in saccade accuracy (pro-saccade task) after the training
[27] Donmez & Cagiltay (2024)	Design-based research study	36 participants (2–18 years, mean age: 11 years) with low vision	To investigate the design processes for developing eye training materials for children with low vision (CLV) using computer game applications based on eye movement tracking to enhance their vision skills	Participation of subjects enrolled, their parents, ophthalmologists, special education teachers, and faculty members from the field of instructional technology and special education	20 min home-based computer game based on eye movement tracking	The training programme may provide objective feedback about the experience of CLV and promote self-training for visual improvement anywhere and anytime
[24] Perra et al. (2020)	Study protocol feasibility	Very preterm (VP) infants with corrected age of 12 months (+/− 1 month)	To assess the feasibility of a study protocol for computerised Attention Control Training (ACT) for VP infants	Battery of tests and tools about general cognitive and motor development, their attention, and their social cognition abilities	Task of search of targets during an interactive stimuli presentation contingent on infants’ direction of gaze	Study protocol
[32] Perra et al. (2021)	Randomised trial	12 very preterm infants (12 months +/− 1 month)	To test the feasibility of delivering the computerised Attention Control Training (ACT) to very preterm (VP) infants	Number and duration of participation of VP infants that completed training/control sessions and VP infants’ engagement; acceptability and completion assessments; quality of eye-tracking data and feedback from parents (questionnaire and semi-structured interviews)	The ACT intervention group watched interactive cartoons for at least 240 s on a computer screen connected to an eye tracker which recorded the infants’ eye movements	VP infants engaged in the ACT games and increased their performance during training. All completed most of the tasks with parents’ positive feedback
[48] Puttemans et al. (2025)	Multiple-case study	Three children aged 7, 12, and 13 years with dyskinetic cerebral palsy	To explore the effects of a four-week intensive eye-tracking intervention on children with dyskinetic cerebral palsy	Goal attainment (GAS), AAV Profile: A Continuum of Learning scale; heart rate variability (HRV); Psychosocial Impact of Assistive Devices Scale (PIADS)	4-week intervention with ET divided into two sequential parts of 2 weeks each (intervention phase 1 and intervention phase 2)	A structured, tailored, four-week intensive eye-tracking intervention can yield successful results
[33] Psotta et al. (2023)	Randomised double-blind controlled trial	57 children (9–12 years) with ADHD (experimental group, *n* = 30; control group, *n* = 27)	To investigate the effects of quiet eye training (QET)-based visuomotor intervention on different aspects of attention in children with ADHD	d2-R Test of Attention; Reaction Test of Alertness (RTA) of the Vienna Test System (VTS); the throwing/catching task of the MABC-2 Test; eye movement pattern; catch performance; arm movement time	5-week QT-based visuomotor training (1 session per week, 35 min per session)	Focused attention in children with ADHD can be improved by a short-term QET-based visuomotor intervention
[22] Scherf et al. (2018)	Study protocol	34 adolescents (10–18 years) with ASD	To assess intervention feasibility and effectiveness in improving sensitivity in eye-gaze cues and social visual attention to faces of the proposed serious game	Performance accuracy, gaze shifts between the face and target and non-target objects and the ratio of average gaze time to the target object versus average gaze time to non-target objects Social Skills Improvement System (SSIS); Social Responsiveness Scale, 2nd Edition (SRS-2)	To play an eye-gaze computer game at home for 30 min, 3 times a week	Study protocol
[25] Scherf et al. (2024)	Study protocol for randomised controlled trial	40 participants (experimental group, *n* = 20; control group, *n* = 20) with ASD	To investigate whether the improved behavioural sensitivity to eye-gaze cues was facilitated by increasing visual attention to faces and/or to the target objects of the directed gaze	Behavioural accuracy, eye-tracking metrics of social visual attention	Serious game training based on referential understanding of the observer’s visual behaviour. 30 min sessions at home, 3 times a week over 10 weeks	Study protocol
[41] Shamir et al. (2023)	Conference article	18 children (5–9 years) with ASD	To present a new technological intervention’s impact on eye contact	Eye contact duration time for the eyes and face, and total gazing time in seconds (pre- and post-intervention)	Eye contact tasks completed by working with the computer game (C-Me). 30 min sessions twice a week over 3 consecutive weeks (total of 6 meetings)	The technological intervention effectively promoted eye contact among all participants
[50] Sosnowski et al. (2022)	Randomised controlled trial	54 children (4–14 years) with ASD (experimental group, *n* = 25; control group, *n* = 29)	To determine feasibility, acceptability, and efficacy of a video game-based digital therapeutic combining applied behaviour analysis techniques and gaze-contingent eye tracking to target emotion recognition in youth with autism spectrum disorder (ASD)	Ekman-60 emotion recognition test	A 6-week (3–5 sessions per week, 15 min per session) intervention with Lookware™ digital therapeutic	Significant improvements in emotion recognition from pre- to post-intervention
[46] Tang et al. (2022)	Randomised controlled trial (2 × 2 ×2 design)	56 participants with ASD or typically developing	To analyse whether storytelling with social contextual information improves visual attention or gaze direction in children with ASD	Eye-tracker data (total fixation duration, TFD, total visit duration, TVD, total fixation count, TFC) and Trial Making Test	8 sessions across 4 weeks, 2 sessions per week, 30 min per session	Storytelling with social contextual information improved gaze behaviour at faces or eyes in ASD and TD when both assessed with photos displayed on a screen, but not in ASD regarding screen-based videos
[33] Valtr et al. (2023)	Randomised controlled trial	106 participants (8–12 years) with ADHD (experimental group, *n* = 54; control group, *n* = 52)	To investigate the effects of quiet eye training (QET) on the neuropsychological functioning and fine motor performance of children with attention deficits	Reaction test of alertness, GNG inhibition test, MLS test: right and left hand (neuropsychological tests)	5-week training (five 35 min training sessions, one session per week) based on stimulation of attention and goal-directed gazing while practising in targeting tasks	Notable impact of the intervention on: processing time and inhibitory control, focused attentional state, processing time and attentional engagement, alertness, and potential fine motor precision
[51] Wang et al. (2020)	Randomised controlled trial	35 3-year-old children with ASD (experimental group) and 41 3-year-old typically developing children (control group)	To examine the feasibility of GCET social attentional training aimed at improving attention to faces and the preliminary efficacy of the training	Participants’ data quality and successful completion of task batteries	SR Eyelink 1000 Plus 500 Hz eye-tracking system and social video displayed on screen. Training duration not specified	The study provides support for training feasibility
[37] Wygnanski-Jaffe et al. (2023)	Prospective, multicentre, randomised, masked, controlled, noninferiority pivotal trial	103 subjects (4–<9 years) with amblyopia	To compare a novel binocular eye-tracking-based home treatment (CureSight; NovaSight, Ltd.) with patching in improving visual outcomes	Amblyopia Treatment Study diplopia assessment, CTS software, E-ETDRS, Randot Preschool Stereoacuity test and a symptom survey	CureSight treatment uses combined anaglyph glasses and an ET. Participants used the device for 90 min/day, 5 days per week for 16 weeks (120 h). The patching group received 2 h of patching 7 days/week (224 h)	The intervention is not inferior to patching and has higher rates of parent preference
[38] Zhu et al. (2023)	Pilot study	34 participants (4–9 years) with unilateral anisometropic amblyopia	To assess visual acuity (VA) and stereoacuity (SA) improvements in children with amblyopia treated with either binocular dichoptic treatment or patching treatment	Electronic Early Treatment Diabetic Retinopathy Study (E-ETDRS) protocol for distance visual acuity (DVA); Pediatric Eye Disease Investigator Group (PEDIG); Amblyopia Treatment Study (ATS) near-acuity test for near visual acuity (NVA); Titmus stereoacuity test for stereoacuity	Group 1: full treatment group using CureSight device for 90 min per day, 5 days a week for 12 weeks. Group 2: part-time treatment group with CureSight 3 days a week. Group 3: patching treatment group (PTG; *n* = 14) wore a patch over the dominant eye 2 h per day, 7 days per week for 12 weeks	VA and SA after binocular dichoptic treatment produced a similar therapeutic outcome to patching
