# Supplementary material for: Eye Tracking for Rehabilitation and Training in Paediatric Neurodevelopmental Disorders: A Systematic Review
Source: Brain Sci. 2026 Mar 21;16(3):337. doi: 10.3390/brainsci16030337 (PMC13023956; doi:10.3390/brainsci16030337)
Supplement: Supplementary file 1 [file brainsci-16-00337-s001.zip › Supplementary Table S1.pdf]

Supplementary Material

Eye Tracking for Rehabilitation and Training in Paediatric Neurodevelopmental Disorders: A Systematic Review

Guido Catalano [1,2], Sara Abbondio [2], Roberta Nicotra [1,2,*], Valentina Berselli [1], Marta Guarischi [3], Valentina Vezzali [2] and Sabrina Signorini [2]

[1] Department of Brain and Behavioral Sciences, University of Pavia, Via Agostino Bassi 21, 27100 Pavia, Italy
[2] Developmental Neuro-Ophthalmology Unit, IRCCS Mondino Foundation, Via Mondino 2, 27100 Pavia, Italy
[3] Unit for Visually Impaired People, Istituto Italiano di Tecnologia, Via Enrico Melen 82, 16100 Genova, Italy
* Correspondence: roberta.nicotra01@universitadipavia.it

Supplementary Table S1: Search builders according database, limited for the last 20 years of publication.

Data base	Search strategy
Pubmed	(("Eye-Tracking"[Title/Abstract] OR ("Eye-Tracking"[Title/Abstract] OR "eye tracker"[Title/Abstract] OR "Oculomotor"[Title/Abstract] OR "eye gaze"[Title/Abstract])) AND ("Training"[Title/Abstract] OR "Rehabilitation"[Title/Abstract] OR "game"[Title/Abstract] OR "gaming"[Title/Abstract] OR "stimulation"[Title/Abstract] OR "Intervention"[Title/Abstract]) AND ("child*"[Title/Abstract] OR "infant*"[Title/Abstract] OR "adolescent*"[Title/Abstract])) AND (2005:2025[pdat])
Scopus	TITLE-ABS (("Eye Tracking" OR "Eye-Tracking" OR "Eye tracker" OR "Oculomotor" OR "Eye Gaze") AND ("Training" OR "Rehabilitation" OR "game" OR "gaming" OR "stimulation" OR "Intervention") AND ("child*" OR "infant*" OR "Adolescent*")) AND (LIMIT-TO (LANGUAGE , "English"))

Web of Science	AB=((((Eye AND Tracking) OR (Eye-Tracking) OR (Eye AND Tracker) OR (oculomotor) OR (eye AND gaze))) AND ((training) OR (rehabilitation) OR (game) OR (gaming) OR (stimulation) OR (intervention)) AND (((child*) OR (infant*) OR (adolescent*))))
